# Student Life in Edinburgh

**Published:** 1889-05-11

**Authors:** A. G. B. Atkinson


### Student Life in Edinburgh.

By A. G. B. Atkinson, B.A.

Readers of *Punch* may remember a drawing which appeared in that paper some time back, in which the vigorous pen of the artist had sketched the portrait of the typical medical student of a bygone generation, whilst opposite was drawn his hard-working successor of to-day. "Look on this picture and on that!" The one lolled in an easy-chair with his feet gracefully resting on the mantelpiece, upon which stood a skull with a clay pipe stuck in its mouth, whilst a jug of beer foamed on the table by his side; the whole a very incarnation of rowdyism and wasted opportunities. How different the other picture, in which is depicted the hard-working student of to-day amidst a pile of books, engaged in the study of anatomy and other kindred subjects; the skull is there, too, but the base uses to which it had been perverted by the first are abandoned, and the article is performing its proper functions as the instructor of the would-be Æsculapius.

"Nous avons changé tout cela"; and the artist merely gave expression to a change which has been working amongst medical students as well as in other places.

Yet, though the improvement has begun, there remains still something to be accomplished in the way of decency and order amongst medical students, as few will be found to deny.

Let the average medical student serve as a type of the many. The day has passed when, as tradition affirms, it was the custom for the embryo physician to spend half the year at his employment, in order to gain sufficient money to keep him at the University for the second half. As a rule, he is born of parents of modest means, not particularly well up in the social scale, and comes to the modern Athens with the definite purpose of passing his examinations in the shortest possible time. This, in Edinburgh, is four years; though, should he fail in either of the three examinations—*i.e.*, the first and second professional and the final—the duration of his course will probably be extended to five years. At the very commencement of his career, unless his relations live in the city, he is thrown utterly upon his own resources, and he takes, probably, a modest lodging in the Meadows, the student's quarter in Edinburgh, corresponding to the Latin quarter in Paris. No one cares how the beginner employs his spare time; whether he spend it in pleasure or in work, in vice or in the seclusion of his chambers, it is all one to the University authorities. What wonder, then, if the boy—for he is nothing more sometimes—falls away from the stern path of duty, and becomes what is technically known as a "waster"? His life is totally different from that of the undergraduate of Oxford or Cambridge, who is looked after by his college authorities, and bound to be within gates by a certain hour.

Again, the social life which forms such a pleasing feature of the "'Varsities," is wanting to the Edinburgh student. This social life receives considerable impetus, owing to the system of living together within college walls and dining in the common hall. Of all this the Edinburgh student knows nothing. He is essentially a solitary individual, and has yet to learn to appreciate the virtue of the after-dinner cup of coffee.

No doubt the formation of a Union Club, on the lines of those of Oxford and Cambridge, may tend to remove this evil. The building for such a club is already erected. It contains spacious reading, writing, and smoking rooms, and will be open for the enrolment of members by the end of the year. Here there will be opportunities for indulgence in some of those social pleasures which help to make life worth living, and for the cultured leisure of Aristotle; and surely some of this culture, some of this refinement, is necessary to the man who is to spend his whole life in ministering to the wants of the sick. That is the very occupation which, more than any other, needs gentleness and refinement.

Truth to say, there is somewhat too much roughness in the modern medical student, though he has improved of late in this respect. The work, itself, no doubt, tends to engender a hardness of character; familiarity, as we all know, breeds contempt. But let it be borne in mind that the perfect doctor is also of necessity the perfect gentleman. The young student should aim, above everything else, at making himself a thorough gentleman, and if he succeeds he will have one of the most important requisites of the perfect physician. Still, it must not be supposed that the Edinburgh student is altogether deprived of social enjoyments. Out of working hours his most popular amusement in winter is football. Matches are numerous, and the excitement over them rages high. Sports, too, have their fair share of attention, but cricket, alas, is a pastime too little patronised in Scotland, though there is a cricket-ground belonging to the University.

Undoubtedly the opportunities for outdoor recreation are scanty, which is, perhaps, an unavoidable evil, the examinations being hard to pass and the examiners ruthless in plucking the insufficiently unprepared. Next to the examination for the London M.D., the Edinburgh medical examinations are stiffer than any in the kingdom, so Northern students have their work before them. Yet who would have it otherwise? In this enlightened age we may fairly demand that the men to whom we are going to entrust our lives, and the lives of those dearest to us, should be acquainted with all the researches of modern science.

For the testing of knowledge the modern system of examination is at least as good as any that can be formulated at the present day, and the numbers who enter at every examination are remarkable. As many as two hundred will present themselves for the final medical in May, the successful in which will obtain their degree of M.B.C.M., and are fully qualified medical practitioners. These candidates and students come from all parts of the world, though mainly from England. Altogether, there are some nineteen hundred, and though there are arts courses as well as medical, the majority adopt the latter.

The facilities offered for the study of medicine are numerous and certainly of the best. Able lecturers and tutors, spacious dissecting-rooms, and an admirable museum are provided; although one fault of the university system seems to lie in the size of the classes, which consist sometimes of as many as four hundred students. As all of these do not work with particular ardour, but delight in the noise and horseplay of youth, it may easily be imagined that the lecturer frequently has no easy task in drilling his somewhat unruly crew. The greater part of the work to be prepared seems to be done not in the lecture theatre, but at home in the evenings.

Having followed our typical young student so far, let us suppose him safely through his three examinations already mentioned. The question next presents itself to him: How is he to make a start in his profession? The most satisfactory way is to obtain, if he can manage to do so, the post of house surgeon or house physician in the infirmary. This is an honorary position, and lasts for six months; the house surgeon lives at the infirmary, and thus gains many opportunities for studying cases which otherwise might have escaped his attention. Supposing the newly-fledged medico to have been six months upon the medical side, he may then spend another six months in the surgical department, after which he is launched upon the world to fight his own way.

The medical profession is overstocked, like every other in this country; still the best man always comes to the front in the end, and all the young student can do is to work hard, make the most of his opportunities, and in no place can these be found in greater abundance than at Edinburgh.

